# Treatment for complete bilateral duplex kidneys with severe hydronephrosis and ureterectasis of the upper moiety in a child: A case report and literature review

**DOI:** 10.3389/fsurg.2022.1019161

**Published:** 2022-11-02

**Authors:** Chengchuang Wu, Fengming Ji, Huangchenghao Zhang, Zhigang Yao, Li Li, Bing Yan

**Affiliations:** ^1^ Department of Urology Surgery, Kunming Children's Hospital, Kunming, China; ^2^ Yunnan Key Laboratory of Children's Major Disease Research, Yunnan Province Clinical Research Center for Children's Health and Disease, Kunming, China

**Keywords:** child, duplex kidney, renal malformation, ureterostomy, ureteroureterostomy

## Abstract

**Aim:**

To explore the treatment experience of the duplex kidney.

**Method:**

A case of the complete bilateral duplex kidney with severe hydronephrosis and ureterectasis in the upper moiety of the kidney diagnosed in the Department of Urology of Kunming Children's Hospital from 2021 to 2022 was retrospectively analyzed and relevant literature was reviewed.

**Results:**

A 2-month-old baby girl was admitted to the hospital because of hydronephrosis of bilateral kidneys found by prenatal ultrasound for 3 months and fever for 3 days. After being given the relevant examinations, the girl was diagnosed with complete bilateral duplex kidneys with severe hydronephrosis and ureterectasis in the upper moiety, and urinary tract infection. The patient's urinary tract infection was poorly controlled after positive anti-infective therapy, so a bilateral ureterostomy was performed. After the surgery, urinary tract infection was soon cured. A bilateral ureteroureterostomy was performed 13 months later, and the patient recovered after 7 days.

**Conclusion:**

Cutaneous ureterostomy combined with late ureteroureterostomy for children with complete bilateral duplex kidneys with severe hydronephrosis in the upper moiety and ureter are not only beneficial to caregivers’ nursing after the operation, but also have significance for salvaging renal function.

## Background

Duplex kidney is a common disease in pediatric urology. There are many controversies due to the children's deformities and treatment methods are diverse and complicated (1). We report a case of severe hydronephrosis and ureterectasis in the upper moiety of bilateral duplex kidney with ectopic ureter on the left side and ureterocele on the right side, and summarize the characteristics of the disease for improving the understanding of pediatricians of the disease.

## Clinical material

### Patient information

The patient's parents informed and agreed to this case report: A 2-month-old baby girl was admitted to the hospital because of hydronephrosis of bilateral kidneys found by prenatal ultrasound for 3 months and had a fever for 3 days. The child was found to have bilateral hydronephrosis in her mother's late pregnancy, and ultrasound was not regularly performed after birth. The child had a fever for 3 days without an obvious cause, and the highest temperature was 39°C. After treatment in a local hospital, the child still had a recurrent high fever. After being referred to Kunming Children's Hospital, the child was given relevant examinations.

### Physical examination

No positive signs were found.

### Laboratory examination

Urine test revealed white blood cell: +++, white blood cell enzyme (+), nitrite (+). Urine culture: *Escherichia coli*.

### Imaging examination

**Figure 1:**
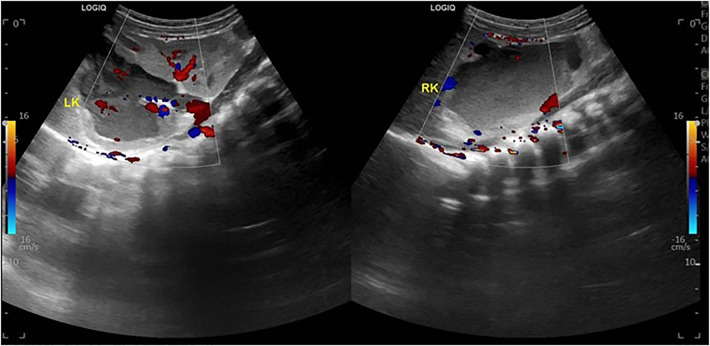
Preoperative ultrasound showed severe hydronephrosis and ureterectasis of bilateral upper moiety.

**Figure 2:**
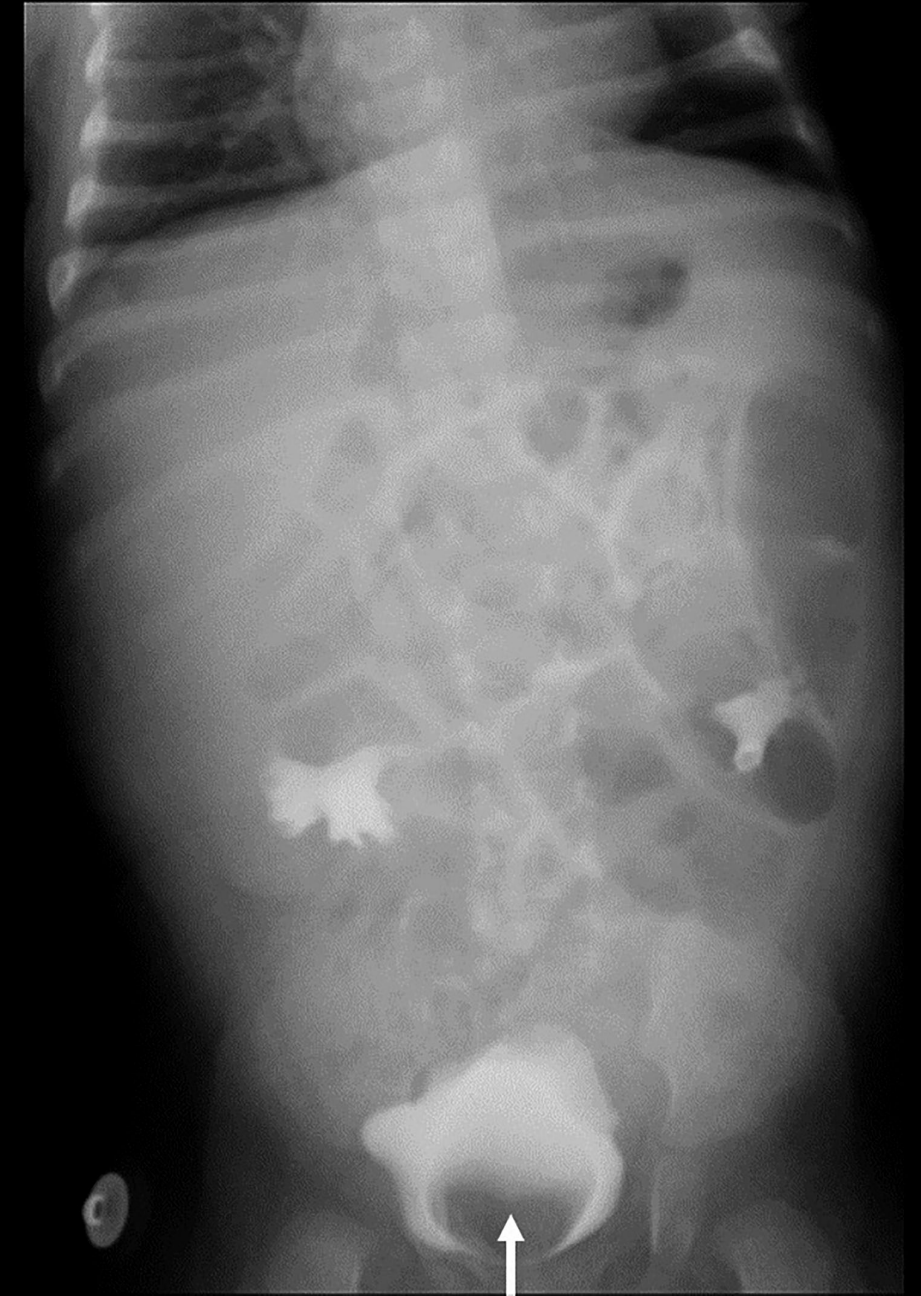
Preoperative IVP showed an ureterocele in the bladder (↑).

**Figure 3:**
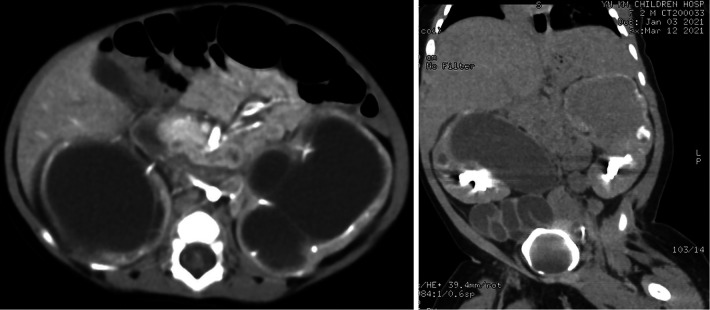
Results of preoperative CT showed severe hydronephrosis and ureterectasis of bilateral upper moiety, left ureteral opening, and the right ureterocele.

Ultrasound of urinary system showed bilateral duplex kidney (Figure 1). Severe hydronephrosis and ureterectasis were found in the bilateral upper moiety. Intravenous pyelography (IVP) showed there was an ureterocele in the bladder (Figure 2). Computerized tomography (CT) revealed severe hydronephrosis and ureterectasis of bilateral upper moiety, left ureteral opening was ectopic in location, and the right ureterocele was found (Figure 3).

**Figure 4:**
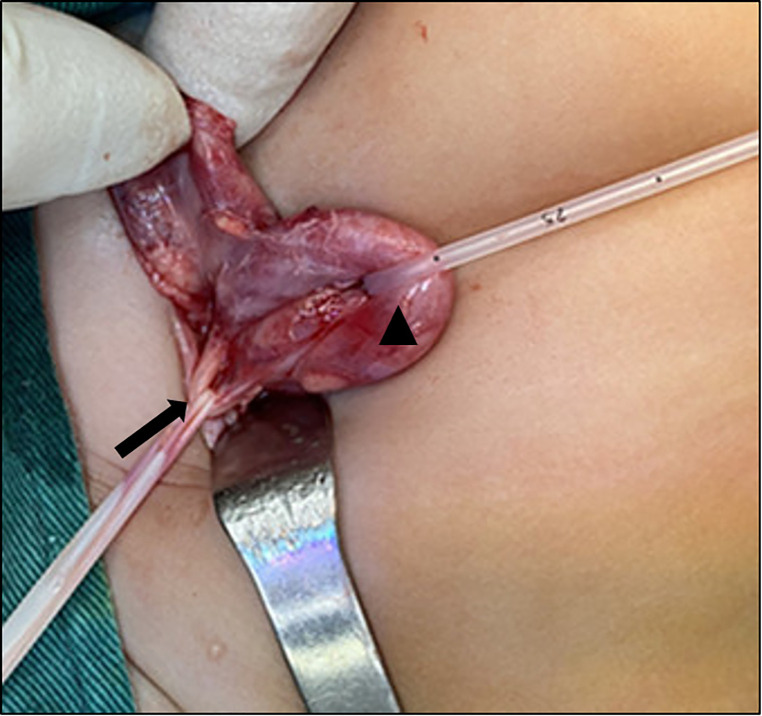
▴: Upper moiety ureter, ↑: lower moiety urtter.

**Figure 5:**
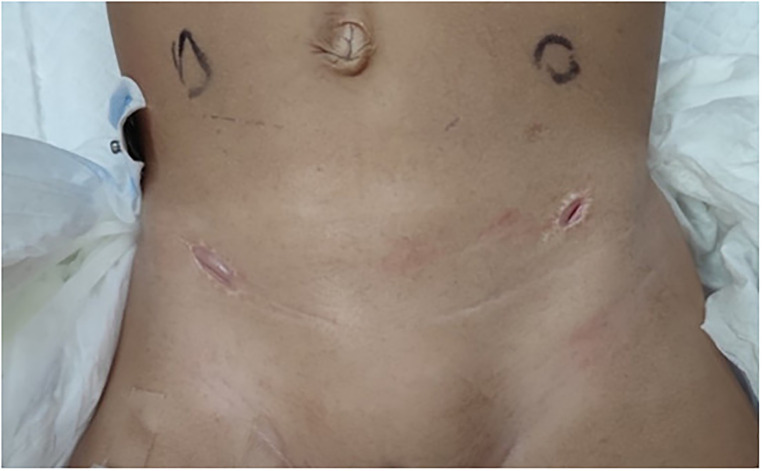
Patient's situation after bilateral cutaneous ureterostomy.

After discussion, cystoscopy and bilateral cutaneous ureterostomy was performed for the patient (Figure 4). In cystoscopy, ectopic insertion of lift upper moiety ureter and ureterocele of right upper moiety ureter were found. The patient's urinary tract infection (UTI) was cured soon after bilateral cutaneous ureterostomy, and she was discharged 5 days later (Figure 5).

**Figure 6:**
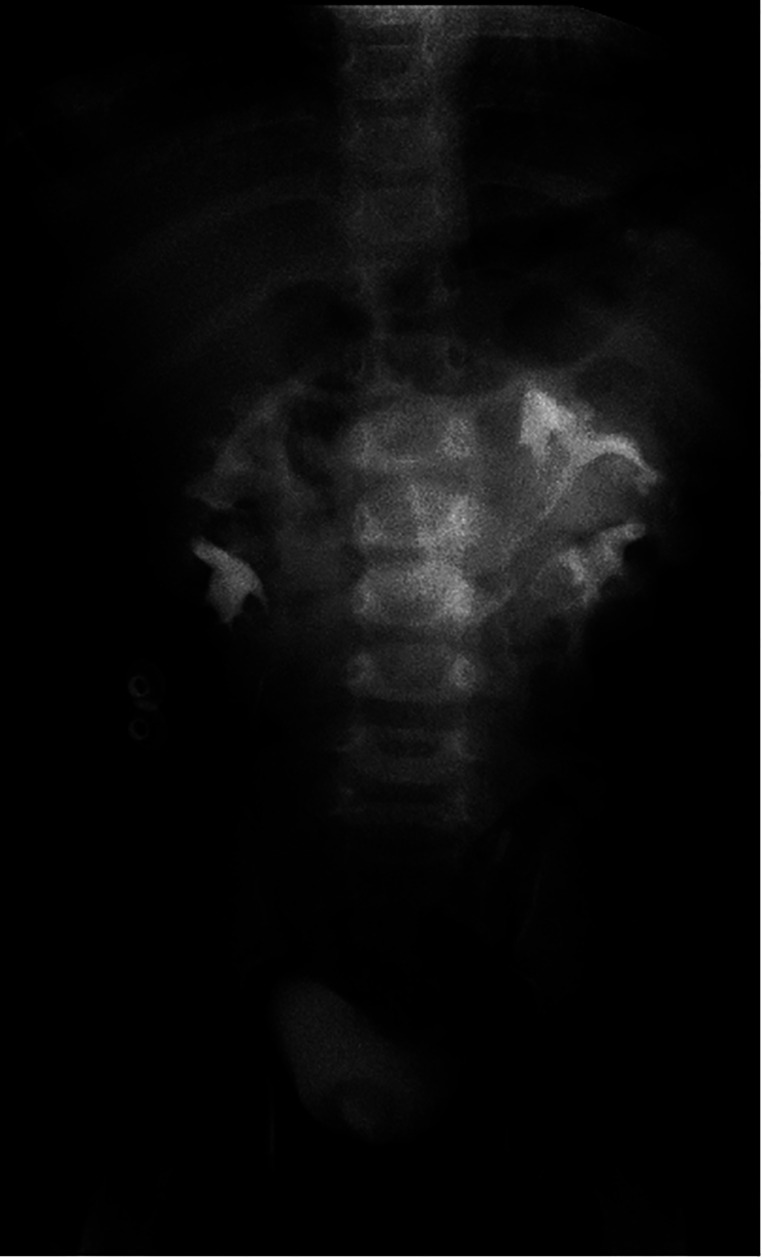
Result of IVP after ureterostomy showed no hydronephrosis in bilateral duplex kidney.

**Figure 7:**
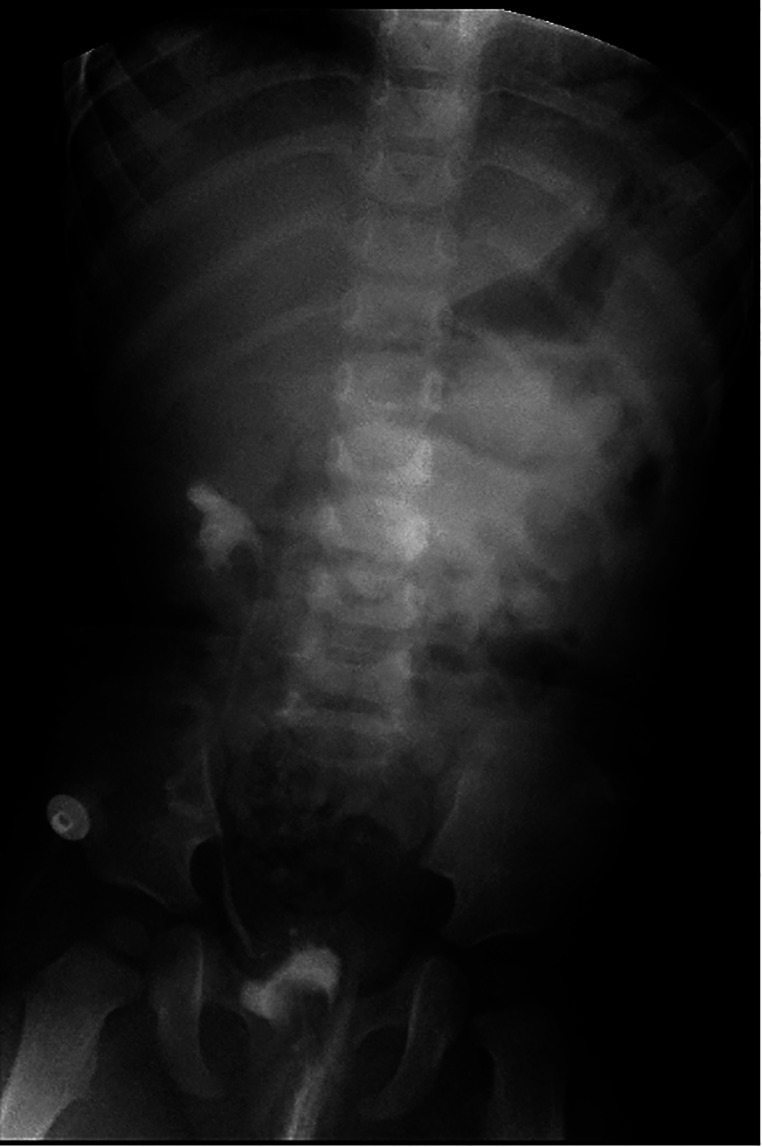
Result of VCU before ureteroureterostomy showed mild vesicoureteral reflux (VUR) on the right lower moiety ureter.

**Figure 8:**
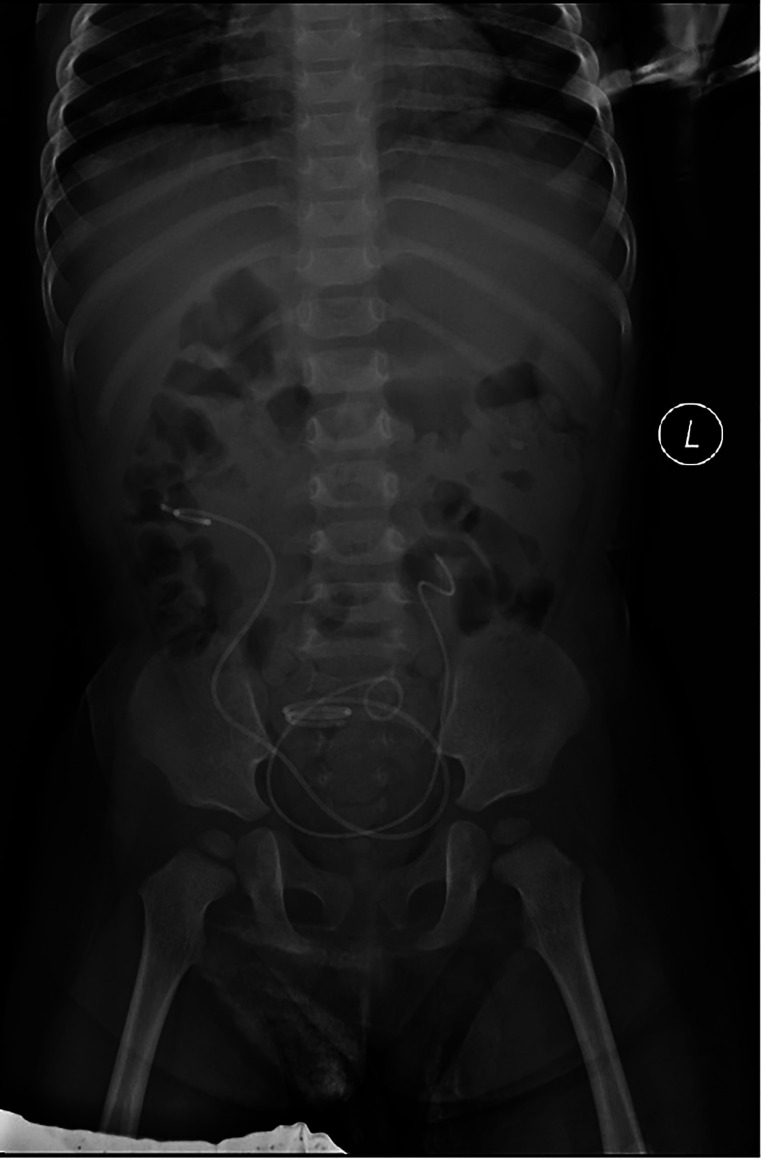
Postoperative situation of the D-J tubes.

No UTI a Figure 5 fever occurred in the patient during discharge. The results of routine urine examination were normal, and ultrasound indicated that bilateral hydronephrosis was relieved obviously. 13 months after the ureterostomy, the child was returned to the hospital for further surgery. IVP showed no hydronephrosis in bilateral duplex kidney (Figure 6). Voiding cystourethrography (VCU) showed mild vesicoureteral reflux (VUR) on the right lower moiety ureter (Figure 7). The patient recovered and was discharged on the 7th day after bilateral ureteroureterostomy. The D-J tubes were removed 1 month after operation (Figure 8).

## Discussion

Prenatal ultrasound is necessary for both pregnant and fetus. With the continuous development and maturity of ultrasound technology, it not only helpful for diagnosing an obstruction of the urinary tract in pregnancies, but also meaningful for permitting the prenatal anatomical assessment of most congenital anomalies of the kidney and urinary tract during the second or third trimester (2, 3). The incidence of duplex kidney malformation is about 0.8% and it is common to women (4, 5). Among children diagnosed with hydronephrosis before birth, 5%–7% are due to duplex kidney (6). The duplex kidney can be divided into incomplete type (Y type) and complete type. The incidence of complete ureteral deformity is about 0.2% (7). According to Weigert-Meyer-Rule, the upper pole is normally seen as ectopic and therefore dysplastic due to obstruction, whereas the lower pole is related to vesicoureteral reflux (8–10).

Fetal interventions include ultrasound-guided bladder puncture and drainage and transurethral incision of ureterocele under fetal cystoscope. Although the decompression effect is good, there are risks of premature rupture of membranes, premature delivery, infection, bleeding, and fetal death, which need to be carefully evaluated and considered (11, 12). Nearly 60% of patients with duplex kidney are asymptomatic and need no treatment after birth. Patients with ectopic ureteral opening, ureteroceles, hydronephrosis, calcuil, urinary tract infection, or non-functional need surgical treatment (13). Treatment for duplex kidney needs to be individualized, and hemi-nephroureterectomy is the earliest technique used in duplex kidney therapy. If the duplex kidney function is good and there is no dysplasia, the upper moiety can be reserved. Common surgical methods include ureteric reimplantation, ureteroureterostomy, pelopureteroplasty, etc. (14). The objective of treatment is to prevent urinary tract infection, and renal damage and to achieve urinary continence (15).

The surgical indication for hemi-nephroureterectomy is upper renal function <10% due to recurrent urinary tract infection with vesicoureteral reflux, or severe obstruction (16, 17). And most scholars agree that upper moiety resection should be performed in patients with recurrent urinary tract infection and ipsilateral abdominal pain (18). However, it is controversial whether the operation should be performed in patients with renal function <10% and no relevant clinical symptom. Proponents believe that surgical removal of non-functional or dysplasia renal tissue and ureters can prevent the long-term occurrence of hypertension or pyelonephritis (1, 19). On the contrary, the possibility of long-term hypertension and pyelonephritis in the non-functional kidney is low, and the operation will easily affect the blood supply and function of the lower kidney, leading to kidney loss (15, 20, 21).

Ureteroureterostomy is usually performed at the level of the iliac vessels to avoid dissociation of the colon, interference with the nerves and vessels of the bladder, and extensive dissociation of the ureter (22). The procedure is not only easier than common sheath ureteral reimplantation but also can protect bladder function from damage. Anastomotic fistula, anastomotic stenosis, and ureteral stump complications are the main postoperative complications of this procedure, and the overall incidence is similar to that of common sheath ureteral bladder replantation (23).

In duplicated ureters with VUR, without obstruction, and with the preserved function of both renal moieties, the gold standard surgical intervention is ureteral reimplantation. However, the incidence of surgical complications is as high as 10%–12.5%, and bladder function will inevitably be disturbed. Studies showed that about 10% of patients require a second surgery (21, 24).

For patients with severe hydronephrosis combined with severe urinary tract infection and sepsis, timely removal of obstruction and urine drainage are beneficial to the recovery of duplex kidney function and the retention of the kidney (25, 26). Common urine drainage methods include ① Pyelostomy: This method is simple and effective. But it requires indent and regular replacement of external drainage tube, and later nursing is relatively tough for patients with troublesome for the caregivers. ② Transurethral incision of ureterocele (TIU): TUI has an invasive, cosmetic, and no external drainage surgical effect. However, this method has secondary or aggravating risks of VUR on the affected side, and patients with large cysts may also have postoperative cyst wall prolapse in the urethra. ③ Cutaneous ureterostomy: This operation has a definite curative effect, no external drainage after the operation, and postoperative nursing of infants is relatively convenient.

In this case, considering that both external renal drainage tubes should be placed after pyelostomy, and unilateral TIU has no definite significance for the relief of obstruction on the other side, so bilateral ureterostomy was performed in this case. The patient's hydronephrosis was significantly relieved after cutaneous ureterostomy, and IVP showed that the development of both kidneys was normal after 13 months. It indicated that timely relief of obstruction was of significance to salvage renal function.

At present, it is still controversial whether ureteral bladder reimplantation should be performed for duplex kidney with lower moiety VUR. Due to the low grade of reflux, ureteric reimplantation was not performed in our case for the following reasons: On the one hand, mild reflux has the possibility of self-healing. On the other hand, if the patient's grade of VUR is aggravated or recurrent urinary tract infection occurs, only a single ureteral bladder reimplantation will be required in the future. Compare with common sheath ureteral reimplantation, it requires a smaller bladder capacity, ureter diameter, and bladder mucosal tunnel length, which is not only beneficial to reduce surgical trauma and bladder disturbance, but also has a higher surgical success rate (24).

When indicated, the type of surgery for children with the complicated duplex renal anomaly is based on renal moiety function and lower tract anatomy, and sequential treatment is meaningful to reduce bladder disturbance, reduce surgical trauma and improve the success rate of surgery.

## Data Availability

The raw data supporting the conclusions of this article will be made available by the authors, without undue reservation.
